# Hypofibrinogenemia induced by high-dose tigecycline—case report and review of literature

**DOI:** 10.1097/MD.0000000000022638

**Published:** 2020-10-23

**Authors:** Qiaomei Fan, Wei Huang, Yayun Weng, Xianze Xie, Zheng Shi

**Affiliations:** Department of Pharmacy, The First Affiliated Hospital of Zhejiang Chinese Medical University, Hangzhou, Zhejiang, China.

**Keywords:** adverse event, high-dose, hypofibrinogenemia, tigecycline

## Abstract

**Rationale:**

Extensive off-label use may affect the safety profile of tigecycline. Tigecycline-associated hypofibrinogenemia is potentially life threatening, although the frequency of life-threatening reactions is unknown and their incidence is easily overlooked. We report a case of 2 instances of treatment with high-dose tigecycline, each of which presented with hypofibrinogenemia.

**Patient concerns:**

An 86-year-old male patient was treated twice with high-dose tigecycline and presented with hypofibrinogenemia both times. The decrease in fibrinogen occurred within 3 to 7 days of tigecycline treatment. Other coagulation parameters had slightly prolonged values.

**Diagnoses:**

Coagulopathy and hypofibrinogenemia.

**Interventions:**

We discontinued the tigecycline.

**Outcomes:**

The fibrinogen level normalized within 5 days after the withdrawal of tigecycline. Following 80 days of hospitalization, the patient was transferred to the rehabilitation hospital for further treatment.

**Lessons:**

We suggest routine strict monitoring of coagulation parameters, particularly fibrinogen. Attention should be paid to below-normal fibrinogen levels due to increased bleeding risk and severity of reaction at fibrinogen levels below 1 g/L.

## 1 Introduction

Tigecycline, the first member of the glycylcycline antibiotics group, has positive in vitro activity against most gram-positive and gram-negative microbes and anaerobic organisms.^[1]^ It is active against carbapenem-resistant Enterobacteriaceae^[2]^ and multidrug-resistant (MDR) *Acinetobacter baumannii.*^[2,3]^ Tigecycline is a bacteriostatic agent that inhibits protein synthesis by binding to the 30S ribosomal subunit and preventing aminoacylated tRNA from entering the A site of the ribosome.^[4]^ It was approved for the treatment of complicated intra-abdominal infections, complicated skin and skin-structure infections, and community-acquired bacterial pneumonia (CAP) at a dose of 50 mg twice daily, following a 100 mg loading dose. Due to the scarcity of antimicrobials effective against resistant bacteria, tigecycline has historically been utilized for off-label indications, frequently in combination with other agents; such off-label uses are increasing globally.^[5]^ However, extensive off-label use may affect the safety profile of tigecycline.

Current data suggest that tigecycline may be useful at higher doses, but the safety of these applications remains in question.^[6]^ The Food and Drugs Administration issued a warning of increased mortality risk associated with the use of intravenous tigecycline, the cause of which was unclear. The most common adverse effects of tigecycline are gastrointestinal disorders (nausea 26% and vomiting 18%).^[7]^ A user-reported adverse event profile study using data from the Food and Drugs Administration adverse event reports database from 2004 to 2009 indicated a relatively high incidence of nausea, vomiting, pancreatitis, and increased hepatic function; an infrequent increase in International Normalized Ratio (INR) was also reported.^[8]^ Coagulopathy was rarely reported in initial studies. In a retrospective study analyzing the adverse events of 145 patients with ventilator-associated pneumonia who were treated with tigecycline, 5 patients with abnormal coagulation were observed, three of whom received a combined treatment with cefoperazone/sulbactam.^[9]^ The concomitant antibiotic was associated with coagulopathy.

The manufacturer's prescribing information for tigecycline suggests a relatively high incidence of activated partial thromboplastin time (APTT) and prothrombin time (PT) prolongation. However, the incidence of hypofibrinogenemia is unknown, and clinicians often ignore this adverse reaction. Here, we present a case of hypofibrinogenemia in a patient treated with high-dose tigecycline. Although severe coagulopathy and hypofibrinogenemia have been previously reported during tigecycline treatment,^[10–15]^ our patient was treated with tigecycline twice and in both instances presented with hypofibrinogenemia, which normalized following withdrawal. We reviewed the literature to discuss the characteristics of the adverse reaction. This study followed the Case Report Guidelines (CARE guidelines)^[16]^ with the patient's informed consent.

## 2 Case report

An 86-year-old male patient was intubated and admitted to the intensive care unit (ICU) because of respiratory failure following a traffic accident. He had a history of hypertension and chronic kidney disease, had no history of blood transfusion, and had no hepatitis B infection. He was unconscious and displayed pinpoint-sized bilateral pupils at admission. CT indicated cerebral hemorrhage in the right frontal lobe, and the white matter area and basal ganglia area of both cerebral hemispheres were thought to be ischemic because of cerebral infarction. On day 3 of hospitalization, the patient was weakly responsive to external stimulation and began to open his eyes in response to verbal command. However, he developed an infection, displaying C-reactive protein of 161 mg/L and a temperature of 38.9°C. Cefoperazone/sulbactam was administered empirically, following which his markers of systemic inflammation decreased and his general condition improved. After 20 days of treatment, the patient was awake and able to open his eyes autonomously.

New onset fever occurred on day 28 of hospitalization, and ventilator-associated pneumonia was diagnosed. Sputum cultures identified MDR *Acinetobacter baumannii* that was sensitive only to tigecycline. The patient was prescribed a new antibacterial regimen of high-dose tigecycline (initiated with a 200 mg loading dose, followed by 100 mg every 12 hour) in combination with cefoperazone/sulbactam. Fever subsided 2 days after the antibacterial regimen change, but the patient's fibrinogen levels continued to decline. After 7 days of antibiotic treatment with tigecycline and cefoperazone/sulbactam, his fibrinogen concentration had decreased below the normal level (1.79 g/L). We discontinued tigecycline on day 14 of therapy. Fibrinogen eventually decreased to 1.18 g/L. Notably, no signs of hepatic dysfunction as reflected by liver enzymes and liver function tests were detected before or after beginning tigecycline treatment. Serum alanine transaminase (ALT) level was 14 U/L, serum aspartate transaminase (AST) level was 24 U/L, alkaline phosphatase level was 171 U/L, and total bilirubin level was 13.0 μmol/L. No clinically significant bleeding event was detected at that time. The antimicrobial regimen was then switched to ceftizoxime as signs of inflammation improved. After discontinuation of tigecycline and cefoperazone/sulbactam, fibrinogen levels normalized within 5 days.

The patient was transferred from the ICU to the department of neurology on day 41 of hospitalization. He presented with myasthenia of limbs because of the cerebrovascular accident and displayed a mild fever. After 9 days of antibiotic treatment with ceftizoxime, the patient's condition suddenly deteriorated, with symptoms of hypotension and respiratory failure. The patient was transferred to the ICU and reintubated and given norepinephrine to maintain blood pressure. Concurrently, ceftizoxime was treatment was stopped, and the anti-infective regimen was switched back to high-dose tigecycline (100 mg q12 hours) and cefoperazone/sulbactam. At this time, the coagulation parameters were normal with the exception of the slightly elevated fibrinogen level (4.9 g/L).

**Figure 1:**
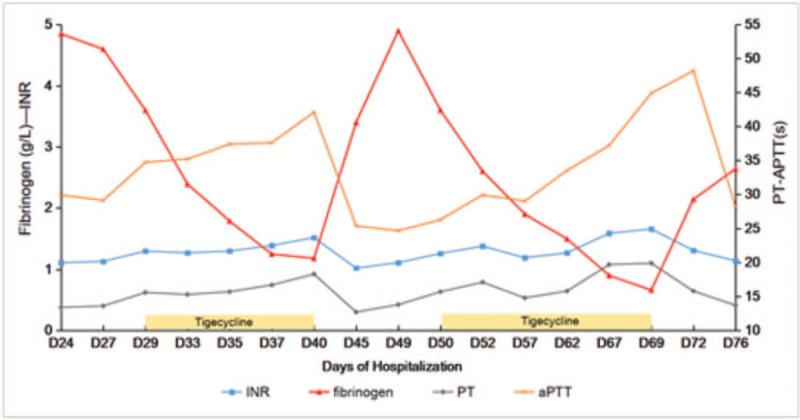
Trends in coagulation lab results during hospitalization. Please note the marked decrease in fibrinogen on days 29 to 40 following tigecycline administration, reversed by its discontinuation (days 41–49). Other coagulation parameters (activated partial thromboplastin time, international normalized ratio, and prothrombin time) had slightly prolonged values. After the second administration of tigecycline during days 50 to 69, coagulation parameters deteriorated again, and fibrinogen level continued to decline.

After 7 days, a slow but progressive decline in fibrinogen was noted, with concurrently elevated PT and INR. We suspected that cefoperazone/sulbactam had an effect on coagulation function, and the antibiotic was, therefore, switched to piperacillin/tazobactam; tigecycline was continued. On day 18 following the start of tigecycline, progressive worsening of hypofibrinogenemia (0.66 g/L) was noted, and prolonged APTT, INR, PT, and thrombin time were observed. The fecal occult blood test was strongly positive. No changes in ALT, AST, and alkaline phosphatase levels were noted, and signs of inflammation were improving (decreased from 104–10 mg/dL). At this point, we strongly suspected an association of fibrinogen decline with the use of tigecycline, and we discontinued the drug. Fibrinogen then improved, and baseline levels were restored (normalized within 5 days, Fig. 1). The patient was transferred to the rehabilitation hospital for further treatment.

This study was approved by the Ethics Committee and institutional review board of The First Affiliated Hospital of Zhejiang Chinese Medical University. Written and informed consent was obtained from the patient for publication of this report.

## 3 Discussion

Tigecycline has a broad spectrum of antibacterial activity. It appears to overcome the major mechanisms conferring resistance to tetracyclines (ribosomal protection and efflux pumps) due to the steric hindrance afforded by its large 9-t-butyl-glycylamido side chain.^[1]^ Bacterial resistance to currently available antibiotics is spreading, but tigecycline remains potently active against MDR bacteria. According to 2018 China Antimicrobial Surveillance Network (CHINET) data, 5% of *Acinetobacter baumannii* isolates were found to be resistant to tigecycline; however, 73.9% were resistant to meropenem. Tigecycline has a long half-life and post-antibiotic effect. The area under the inhibitory curve (AUC/MIC) is considered to most accurately describe the pharmacokinetic and pharmacodynamic properties of a drug^[17]^; accordingly, some authors have found that as dosage increased, tigecycline showed positive pharmacokinetic characteristics and better clinical outcomes.^[18,19]^ A high-dose tigecycline regimen consisting of a 200 mg loading dose and a maintenance dose of 100 mg every 12 hours has been proposed for treating severe infections due to MDR bacteria.^[20]^ Data reported to date suggest that high-dose tigecycline is associated with better outcomes and is well tolerated in the treatment of critically ill patients.^[21]^

The most common adverse effects of tigecycline (nausea and vomiting) had little effect on critically ill patients who were sedated and mechanically ventilated. Instead, clinicians should be alert to the development of hepatic and coagulation dysfunction. Few reports in the literature address tigecycline-associated coagulopathy and hypofibrinogenemia (Table 1).^[10–15]^ The first case report described a patient with end-stage renal disease who received prolonged tigecycline therapy while experiencing a life-threatening coagulation disorder.^[10]^ After 35 days of tigecycline treatment, fibrinogen levels decreased to 0.38 g/L. This may have occurred due to a lack of regular follow up. Fibrinogen recovered within 5 days after tigecycline withdrawal. In addition to a few case reports, four small clinical studies have addressed tigecycline-associated coagulopathy and hypofibrinogenemia.^[22–25]^ Karaiskos et al^[22]^ found that 10% of patients developed hypofibrinogenemia during treatment with standard dose tigecycline, with a median onset of 8 days of treatment. Routsi et al^[23]^ described decreased fibrinogen levels in ICU patients following high-dose tigecycline treatment. Zhang et al^[24]^ evaluated 20 patients treated with tigecycline and found a reduction in fibrinogen levels that was proportional to the dose, not to age. In contrast, Leng et al^[25]^ found that the level of fibrinogen was not significantly influenced by dosage, but patients with more severe illnesses in the ICU were not included in their study.

**Table 1:**
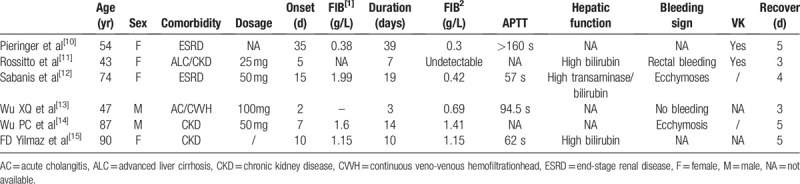
Cases regarding tigecycline-associated hypofibrinogenemia that have been reported.

In our case, the patient received high-dose tigecycline therapy, which was prescribed off label. Before the initiation of tigecycline therapy, fibrinogen level was slightly elevated. On day 7 following the start of tigecycline, a marked progressive worsening of hypofibrinogenemia (0.66 g/L) was noted. The fibrinogen level recovered to within the normal range of 2 to 4 g/L 5 days after tigecycline was discontinued. A similar pattern was observed with the second use of tigecycline. Fibrinogen levels continued to deteriorate as cefoperazone/sulbactam was replaced by piperacillin/tazobactam. However, sepsis contributed to the development of coagulopathy.^[26]^ It is notable, however, that the general condition of the patient and his inflammatory markers were improving at the time of onset of the coagulation disorder. The Naranjo Adverse Drug Reaction Probability Scale^[27]^ score of 10 points categorizes this adverse drug reaction as definite (Table 2), indicating with certainty that tigecycline caused the severe hypofibrinogenemia.

**Table 2:**
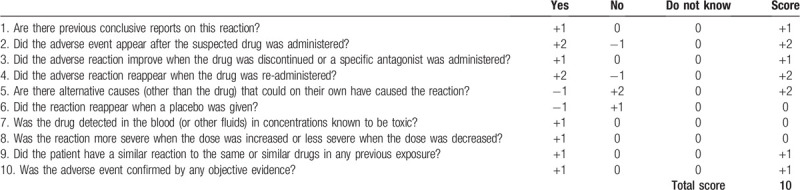
Naranjo Adverse Drug Reaction Probability Scale.

Tigecycline causes coagulation disorders and hypofibrinogenemia, and these adverse reactions display common characteristics. The decrease in fibrinogen generally occurred within 3 to 7 days of tigecycline treatment, and the decrease was sustained throughout the course of the day. The half-life of circulating fibrinogen is approximately 3 to 4 days.^[28]^ Fibrinogen is involved in several important physiological functions, including triggering of platelet adhesion and forming of fibrin polymers in hemostasis and thrombosis. Fibrinogen levels normalized within 5 days after tigecycline withdrawal. Other coagulation parameter abnormalities, including prolongation of APTT and PT, were not consistent with the reduction of fibrinogen, and there was no change in AST, ALT, total bilirubin, or creatinine levels. Therefore, hepatic and renal function could not be correlated with fibrinogen reduction. The influence of tigecycline dosage on fibrinogen levels is still controversial, and both the standard dose and high-dose tigecycline can result in a reduction in fibrinogen levels. A prospective study or a larger sample retrospective study is needed to identify the specific risks inherent to reduction in fibrinogen level.

The main mechanism by which tigecycline induces hypofibrinogenemia is ambiguous. Vitamin K deficiency due to antimicrobial effects on the flora of the intestine is a possible mechanism underlying coagulation disorders, but this can be excluded in some patients who received vitamin K supplementation.^[10,11]^ Fibrinogen is a hexamer composed of 3 polypeptide chains (Aα, Bβ, and γ) that are centrally connected by disulfide bonds.^[29]^ All three polypeptides are synthesized by hepatocytes and assembled into fibrinogen in the liver.^[30]^ Fibrinogen circulates in the plasma at a concentration of approximately 2 to 4 g/L.^[31]^ Reduced synthesis should be examined as a cause of fibrinogen decrease. An impairment in hepatic fibrinogen synthesis can cause reduced levels of fibrinogen, and hypofibrinogenemia has been reported in association with medications that impair the synthetic functions of the liver (eg, L-asparaginase, valproic acid, and possibly allopurinol).^[32–35]^ However, hepatic dysfunction was not implicated in this patient because transaminase and other liver tests were normal; regardless, fibrinogen synthesis is controlled at the transcription level.^[36]^ As an acute-phase reactant, fibrinogen biosynthesis is increased by interleukin (IL)-6-mediated increases in the transcription of the fibrinogen mRNA^[37]^; IL-1 and tumor necrosis factor-alpha suppress fibrinogen synthesis.^[38]^ There is, however, room for speculation that tigecycline may interfere with the production of fibrinogen by influencing the level of cytokines or by directly influencing the transcription of fibrinogen mRNA.

In conclusion, tigecycline may induce coagulation disorders and hypofibrinogenemia. Further study is needed to identify the risks of reduced fibrinogen levels. In light of the increased use of tigecycline due to the emergence of MDR bacteria, we suggest routine, strict monitoring of coagulation parameters, particularly fibrinogen levels. Attention should be paid to below-normal fibrinogen levels because fibrinogen levels below 1 g/L are associated with increased risk and severity of bleeding.

## Author contributions

**Data curation:** Xianze Xie.

**Investigation:** Qiaomei Fan.

**Writing – original draft:** Qiaomei Fan, Wei Huang, Yayun Weng.

**Writing – review & editing:** Zheng Shi.

## References

[1] Mckeage K Keating GM Tigecycline. Drugs 2008;68:2633–44. 19093704 10.2165/0003495-200868180-00008

[2] Fritzenwanker M Imirzalioglu C Herold S Treatment options for carbapenem- resistant gram-negative infections. DtschArztebl Int 2018;115:345–52. 10.3238/arztebl.2018.0345 PMC6172649 29914612

[3] Karageorgopoulos DE Kelesidis T Kelesidis I Tigecycline for the treatment of multidrug-resistant (including carbapenem-resistant) Acinetobacter infections: a review of the scientific evidence. J Antimicrob Chemother 2008;62:45–55. 18436554 10.1093/jac/dkn165 PMC8057304

[4] Pankey GA Tigecycline. J Antimicrob Chemother 2005;56:470–80. 16040625 10.1093/jac/dki248

[5] Conde-Estévez D Grau S Horcajada JP Off-label prescription of tigecycline: clinical and microbiological characteristics and outcomes. Int J Antimicrob Agents 2010;36:471–2. 20828992 10.1016/j.ijantimicag.2010.07.006

[6] Falagas ME Vardakas KZ Tsiveriotis KP Effectiveness and safety of high-dose tigecycline-containing regimens for the treatment of severe bacterial infections. Int J Antimicrob Agents 2014;44:1–7. 24602499 10.1016/j.ijantimicag.2014.01.006

[7] Kaewpoowat Q Ostrosky-Zeichner L Tigecycline: a critical safety review. Expert Opinion on Drug Safety 2015;14:335–42. 25539800 10.1517/14740338.2015.997206

[8] Kadoyama K Sakaeda T Tamon A Adverse event profile of tigecycline: data mining of the public version of the U.S. Food and Drug Administration adverse event reporting system. Biol Pharm Bull 2012;35:967–70. 22687540 10.1248/bpb.35.967

[9] Chen Z Shi X Adverse events of high-dose tigecycline in the treatment of ventilator-associated pneumonia due to multidrug-resistant pathogens. Medicine (Baltimore) 2018;97:1–7. 10.1097/MD.0000000000012467 PMC6160260 30235740

[10] Pieringer H Schmekal B Biesenbach G Severe coagulation disorder with hypofibrinogenemia associated with the use of tigecycline. Ann Hematol 2010;89:1063–4. 20174923 10.1007/s00277-010-0911-7

[11] Rossitto G Piano S Rosi S Life-threatening coagulopathy and hypofibrinogenaemia induced by tigecycline in a patient with advanced liver cirrhosis. Eur J Gastroenterol Hepatol 2014;26:681–4. 24667348 10.1097/MEG.0000000000000087

[12] Sabanis N Paschou E Gavriilaki E Hypofibrinogenemia induced by tigecycline: a potentially life-threatening coagulation disorder. Infect Dis (Lond) 2015;47:743–6. 25951751 10.3109/23744235.2015.1043942

[13] Wu X Zhao P Dong L A case report of patient with severe acute cholangitis with tigecycline treatment causing coagulopathy and hypofibrinogenemia. Medicine (Baltimore) 2017;96:1–3. 10.1097/MD.0000000000009124 PMC5728965 29245350

[14] Wu PC Wu CC Tigecycline-associated hypofibrinogenemia: a case report and review of the literature. ID Cases 2018;11:56–7. 29560313 10.1016/j.idcr.2018.01.003 PMC5857890

[15] Yilmaz Duran F Yildirim H Şen EM A lesser known side effect of tigecycline: hypofibrinogenemia. Turk J Haematol 2018;35:83–4. 29212626 10.4274/tjh.2017.0310 PMC5843784

[16] Gagnier JJ Kienle G Altman DG The CARE guidelines: consensus-based clinical case reporting guideline development. Glob Adv Heal Med 2013;2:38–43. 10.7453/gahmj.2013.008 PMC3833570 24416692

[17] Giamarellou H Poulakou G Pharmacokinetic and pharmacodynamic evaluation of tigecycline. Expert Opin Drug Metab Toxicol 2011;7:1459–70. 21958044 10.1517/17425255.2011.623126

[18] Cunha BA Baron J Cunha CB Once daily high dose tigecycline- pharmacokinetic/pharmacodynamic based dosing for optimal clinical effectiveness: dosing matters, revisited. Expert Rev Anti Infect Ther 2017;15:257–67. 27917692 10.1080/14787210.2017.1268529

[19] Cunha BA Baron J Cunha CB Monotherapy with high-dose once-daily tigecycline is highly effective against *Acinetobacter baumanii* and other multidrug-resistant (MDR) gram-negative bacilli (GNB). Int J Antimicrob Agents 2018;52:119–20. 29501604 10.1016/j.ijantimicag.2018.02.011

[20] Garnacho-Montero J Ferrándiz-Millón C High dose of tigecycline for extremely resistant Gram-negative pneumonia: yes, we can. Critical Care 2014;18:157–8. 25043402 10.1186/cc13942 PMC4075419

[21] De Pascale G Montini L Pennisi M High dose tigecycline in critically ill patients with severe infections due to multidrug-resistant bacteria. Critical Care 2014;18:2–9. 10.1186/cc13858 PMC4057423 24887101

[22] Karaiskos I Ioannidis K Galani L Hypofibrinogenaemia associated with the administration of tigecycline. Conference Paper; 2014.

[23] Routsi C Kokkoris S Karaiskos I High-dose tigecycline-associated alterations in coagulation parameters in critically ill patients with severe infections. Int J Antimicrob Agents 2015;45:90–3. 10.1016/j.ijantimicag.2014.07.014 25241261

[24] Zhang Q Zhou S Zhou J Tigecycline treatment causes a decrease in fibrinogen levels. Antimicrob Agents Chemother 2015;59:1650–5. 25547356 10.1128/AAC.04305-14 PMC4325772

[25] Leng B Xue YC Zhang W A retrospective analysis of the effect of tigecycline on coagulation function. Chem Pharm Bull 2019;67:258–64. 10.1248/cpb.c18-00844 30828002

[26] Semeraro N Ammollo CT Semeraro F Coagulopathy of acute sepsis. Semin Thromb Hemost 2015;41:650–8. 26305237 10.1055/s-0035-1556730

[27] Naranjo CA Busto U Sellers EM A method for estimating the probability of adverse drug reactions. Clin Pharmacol Ther 1981;30:239–45. 7249508 10.1038/clpt.1981.154

[28] Collen D Tytgat GN Claeys H Metabolism and Distribution of Fibrinogen. I. Fibrinogen turnover in physiological conditions in humans. Br J Haematol 1972;22:681–700. 5064500 10.1111/j.1365-2141.1972.tb05715.x

[29] Medved L Weisel JW Recommendations for nomenclature on fibrinogen and fibrin. J ThrombHaemost 2009;7:355–9. 10.1111/j.1538-7836.2008.03242.x PMC3307547 19036059

[30] Tennent GA Brennan SO Stangou AJ Human plasma fibrinogen is synthesized in the liver. Blood 2007;109:1971–4. 17082318 10.1182/blood-2006-08-040956

[31] Levy JH Goodnough LT How I use fibrinogen replacement therapy in acquired bleeding. Blood 2015;125:1387–93. 25519751 10.1182/blood-2014-08-552000

[32] Gralnick HR Henderson E Hypofibrinogenemia and coagulation factor deficiencies with L-asparaginase treatment. Cancer 1971;27:1313–20. 5282618 10.1002/1097-0142(197106)27:6<1313::aid-cncr2820270606>3.0.co;2-w

[33] Whitecar JP Jr Bodey GP Harris JE L-asparaginase. N Engl J Med 1970;282:732–4. 4906449 10.1056/NEJM197003262821307

[34] Hauser E Seidl R Freilinger M Hematologic manifestations and impaired liver synthetic function during valproate monotherapy. Brain Dev 1996;18:105–9. 8733899 10.1016/0387-7604(95)00139-5

[35] Yin ZQ Xu JL Li YQ Transient hypofibrinogenemia due to allopurinol. Drug Des DevelTher 2014;8:1231–3. 10.2147/DDDT.S66868 PMC4159221 25214766

[36] Green F Humphries S Control of plasma fibrinogen levels. Baillieres Clin Haematol 1989;2:945–59. 2688760 10.1016/s0950-3536(89)80053-8

[37] Woods A Brull DJ Humphries SE Genetics of inflammation and risk of coronary arterydisease: the central role of interleukin-6. Eur Heart J 2000;21:1574–83. 10988009 10.1053/euhj.1999.2207

[38] Saliba R Paasch L El Solh A Tigecycline attenuates staphylococcal superantigen-induced T-cell proliferation and production of cytokines and chemokines. Immunopharmacol Immunotoxicol 2009;31:583–8. 19874226 10.3109/08923970902838672

